# LYNSU: automated 3D neuropil segmentation of fluorescent images for *Drosophila* brains

**DOI:** 10.3389/fninf.2024.1429670

**Published:** 2024-07-29

**Authors:** Kai-Yi Hsu, Chi-Tin Shih, Nan-Yow Chen, Chung-Chuan Lo

**Affiliations:** ^1^Institute of Systems Neuroscience, College of Life Science, National Tsing Hua University, Hsinchu, Taiwan; ^2^Department of Applied Physics, Tunghai University, Taichung, Taiwan; ^3^National Applied Research Laboratories, National Center for High-Performance Computing, Hsinchu, Taiwan; ^4^Brain Research Center, National Tsing Hua University, Hsinchu, Taiwan

**Keywords:** fluorescence image, U-net, YOLO, connectomics, image segmentation, anatomical analysis

## Abstract

The brain atlas, which provides information about the distribution of genes, proteins, neurons, or anatomical regions, plays a crucial role in contemporary neuroscience research. To analyze the spatial distribution of those substances based on images from different brain samples, we often need to warp and register individual brain images to a standard brain template. However, the process of warping and registration may lead to spatial errors, thereby severely reducing the accuracy of the analysis. To address this issue, we develop an automated method for segmenting neuropils in the *Drosophila* brain for fluorescence images from the *FlyCircuit* database. This technique allows future brain atlas studies to be conducted accurately at the individual level without warping and aligning to a standard brain template. Our method, LYNSU (Locating by YOLO and Segmenting by U-Net), consists of two stages. In the first stage, we use the YOLOv7 model to quickly locate neuropils and rapidly extract small-scale 3D images as input for the second stage model. This stage achieves a 99.4% accuracy rate in neuropil localization. In the second stage, we employ the 3D U-Net model to segment neuropils. LYNSU can achieve high accuracy in segmentation using a small training set consisting of images from merely 16 brains. We demonstrate LYNSU on six distinct neuropils or structures, achieving a high segmentation accuracy comparable to professional manual annotations with a 3D Intersection-over-Union (IoU) reaching up to 0.869. Our method takes only about 7 s to segment a neuropil while achieving a similar level of performance as the human annotators. To demonstrate a use case of LYNSU, we applied it to all female *Drosophila* brains from the *FlyCircuit* database to investigate the asymmetry of the mushroom bodies (MBs), the learning center of fruit flies. We used LYNSU to segment bilateral MBs and compare the volumes between left and right for each individual. Notably, of 8,703 valid brain samples, 10.14% showed bilateral volume differences that exceeded 10%. The study demonstrated the potential of the proposed method in high-throughput anatomical analysis and connectomics construction of the *Drosophila* brain.

## 1 Introduction

Over the past few decades, neuroscience has evolved from a discipline primarily dependent on experimental biology into an interdisciplinary field of study (Bassett et al., 2020; Vázquez-Guardado et al., 2020). Notably, breakthroughs in imaging applications, such as high-resolution neural imaging techniques, including fluorescent microscopy and electronic microscopy (Hwu et al., 2017; Chu et al., 2019; Lin et al., 2019; Fu et al., 2021; Werner et al., 2021; Fan et al., 2022; Peddie et al., 2022), have been significant. These advancements have enabled us to observe neural tissues and circuits with unprecedented speed and resolution. In this context, analyzing variations in brain structures across different individuals or the expression intensity of genes in various brain regions has become a crucial research direction (Lee et al., 2015; Wolff and Rubin, 2018; Kondo et al., 2020; Mehta et al., 2023). Consequently, developing an automated and high-throughput method for this analysis has become particularly important (Lecoq et al., 2019; Balasubramanian et al., 2023; Qiao et al., 2023).

Indeed, several studies have developed brain region segmentation algorithms for CT and MRI images (Dolz et al., 2018; Roy et al., 2018; Billot et al., 2020; Tan et al., 2020; Ranjbarzadeh et al., 2021; Zopes et al., 2021; Wang T. et al., 2022; Nour Eddin et al., 2023; Wang et al., 2023). However, a similar method for optical images of the *Drosophila* brain has not yet been developed. The *Drosophila* brain contains 58 neuropils (Chiang et al., 2011), and each neuropil, if segmented manually, would take up to 4 h on average. Therefore, manually segmenting every neuropil for each optical image of the *Drosophila* brain is impractical. Instead, a common practice is to warp and align optical brain images obtained from different individuals to a standard brain template (Jefferis et al., 2007; Lin et al., 2007), which contains segmented brain regions, and perform the subsequent analysis in the warped brain images. While this method facilitates subsequent statistical analysis and interpretation, it also introduces spatial errors (Peng et al., 2011; Bogovic et al., 2020; Court et al., 2023). These inaccuracies could impact the precise understanding of neural circuits, thereby reducing the reliability of research findings. To address this issue, one should segment neuropils directly in the original images to avoid errors introduced during the alignment process.

Developing more accurate and efficient image analysis algorithms becomes exceptionally crucial. This involves improving the accuracy of segmentation and alignment and considering computational costs and time efficiency to meet the needs of large-scale, high-throughput studies. Simultaneously, segmenting neuropils directly from the original data can avoid errors introduced during the alignment process, thus enhancing the accuracy and reliability of the analysis.

The present paper introduces a novel computational method, LYNSU (Locating by YOLO and Segmenting by U-Net), specifically designed for segmenting neuropils in *Drosophila* brain fluorescence images. Our method is divided into two stages based on a detection-led segmentation workflow. In the first stage, we detect the location of the neuropil of interest using the YOLOv7 (Wang C. Y. et al., 2022) model, renowned for its exceptional inference speed and rapid convergence during training. These characteristics make YOLOv7 particularly suitable for high-throughput tasks required for large databases like *FlyCircuit* (Chiang et al., 2011), significantly reducing computational time for detecting neuropils. In the second stage, we segment the neuropil in the bounding box defined by YOLOv7 using the 3D U-Net (Ronneberger et al., 2015; Iakubovskii, 2019; Solovyev et al., 2022) model, a deep learning model specifically designed for three-dimensional image segmentation.

The innovation of this method lies in its combination of advanced object detection technology with specialized image segmentation algorithms, achieving efficient and accurate segmentation. Through LYNSU, we anticipate more in-depth and detailed analyses of *Drosophila* brain structures, bringing new insights to neuroscience. This study demonstrates the potential applications of computational methods in biomedical image analysis and provides a reliable reference model for future high-throughput image analyses.

## 2 Materials and methods

### 2.1 Dataset

The 3D fluorescence image data we used in this study were obtained from the *FlyCircuit* database,^1^ which hosts images from 28,573 *Drosophila* brains. These *Drosophila* brain images were acquired using high-resolution confocal microscopy. Each image contains a channel of GAL4 signals for individual neurons and a channel of anti-DLG staining for neuropils, and the signal of each voxel is represented by a value ranging from 0 to 255. The calibrated voxel size of the images is x:y:z = 0.32 × 0.32 × 1 μm, and the XY plane resolution is 1024x1024.

Two datasets were created from the *FlyCircuit* database for training purposes. For the first dataset, we randomly selected 1000 3D images from the *FlyCircuit* database to train the YOLOv7 model. We used the Labelimg tool to annotate the neuropil bounding boxes, with 440 images going through this annotation process. Afterward, we randomly selected 400 of these images to serve as the training set, while the remaining 40 images were used as the test set.

The second dataset was primarily prepared for the training of the 3D U-Net model. We randomly extracted 18 Drosophila brain images from our database, each representing a different type of Gal4 Driver. This approach ensured that our study’s dataset encompassed a variety of brightness characteristics. Next, the six target neuropils or structures, AL, MB, CAL, FB, EB, and PB, in the 18 brains were manually segmented by trained annotators using the ZEISS arivis Cloud tool. On average, the annotation of each neuropil took about 4 h, meaning that we invested approximately 432 h in this annotation work. We specifically requested that multiple annotators label the same brain to ensure consistency among multiple annotators. We verified the consistency of the human annotation and found that the 3D IoU scores among different annotators for the same brain reached 0.85, effectively ensuring the quality and consistency of our annotation work.

### 2.2 Neuropils detection and localization

We adopted a two-stage model training strategy to achieve superior neuropil segmentation effects and efficient computational speed. Focusing on smaller Regions of Interest (ROIs) for segmentation significantly reduces the computational load, leading to faster processing times and improved accuracy. This approach allows us to maintain high-resolution details while enhancing speed and performance. This strategy significantly enhances the accuracy of segmentation and effectively reduces the GPU computational resource consumption. We used the NVIDIA A100 40GB GPU in the first stage for model training. These images were projected into 2D images on the XY plane using two different projection methods: summation of brightness along the Z-axis and maximum brightness value along the Z-axis. We trained the YOLOv7 model on the training set (400 images from the first dataset) through 100 iterations. On the test set (40 images), this model performed excellently, achieving a mean Average Precision (mAP)@0.5 of 0.9955 and within the range of mAP@0.5:0.95 (different IoU thresholds from 0.5 to 0.95 in steps of 0.05), it scored 0.8489. Note that due to spatial overlap between neuropils or structures, some of them share the same ROI. Specifically, MB and CAL share the same ROI, while FB, EB, and PB share the central complex’s ROI, and the AL neuropil has its own ROI. Therefore, in the recent study, we only train YOLOv7 to detect three different ROIs.

Next, we used YOLOv7 to detect the remaining 560 images that were not involved in the training. We visually inspect the result and label detection as a success if the resulting ROI (bounding box) covers the entire neuropil. YOLOv7 achieves a high success rate of 99.28%, and it takes only about ten milliseconds to detect a neuropil in each image.

### 2.3 Neuropils segmentation

For neuropil segmentation, we had 18 manually segmented brains (the second dataset) as the training and test set. We first used YOLOv7 to detect the ROIs from these brains. Subsequently, we extracted the corresponding 3D images from the ROIs. For effective data augmentation, we implemented zero-padding along the Z-axis, extending it to 124 layers while downscaling the XY plane by reducing the resolution of the XY plane to 168 × 168.

We adopted an overlapping sliding window strategy for data augmentation by setting the window size to 64x128x128, with a sliding distance of 20 each time. We performed a mirroring process on the Z-axis to further increase data diversity. Taking the MB as an example, we generated 144 cubes for each set of neuropils, meaning that 16 brains can produce 2304 cubes as the training data for the 3D U-Net. We independently trained a 3D U-Net model for each unique neuropil in the second stage. The model’s architecture featured a robust six-layer ResNet-50 (He et al., 2015) for both the encoder and decoder, enhancing the network’s feature extraction capabilities. To manage the spatial dimensions effectively, the network underwent five rounds of MaxPooling3D for downscaling and an equal number of UpSampling3D operations for reconstruction, ensuring detailed and accurate segmentation outputs. The final layer of the network utilized a softmax activation function to classify each voxel accurately. The learning rate of these models was set to 0.0001, and Adam was chosen as the optimization algorithm. Regarding loss function selection, we adopted a composite loss function, combining Dice loss and Categorical Focal Loss.

Dice loss is a function specifically designed for image segmentation problems and particularly suitable for scenarios with class imbalances. The mathematical formula for Dice loss is:


$$\mathrm{Dice}\,\mathrm{Loss}=1-\frac{2\times \left|{\text{Y}}_{\text{true}}\,⋂{\text{Y}}_{\text{pred}}\right|}{\left|{\text{Y}}_{\text{true}}\right|+\left|{\text{Y}}_{\text{pred}}\right|}$$

where Y_true_ is the set of ground truth labels and Y_pred_ is the set of labels predicted by the model. This formula can also be represented as:


$$\mathrm{Dice}\,\mathrm{Loss}=1-\frac{2\times \text{TP}}{2\times \text{TP}+\text{FP}+\text{FN}}$$

where TP represents true positive, FP false positive, and FN false negative. We set the weight ratio for the background and neuropils in the Dice loss as 0.4 and 0.6, respectively. We further incorporated weighting on category by setting the background weight *w*_1_ and neuropil weight *w*_2_. Thus, the weighted Dice loss function can be expressed as:


Weighted⁢Dice⁢Loss=

$$1-\frac{2\times \left(w_{1}\times {\text{TP}}_{1}+w_{2}\times {\text{TP}}_{2}\right)}{\left(w_{1}\times {\text{TP}}_{1}+w_{2}\times {\text{TP}}_{2}\right)+\left(w_{1}\times {\text{FN}}_{1}+w_{2}\times {\text{FN}}_{2}\right)}$$

$$\;\;+\left(w_{1}\times {\text{FP}}_{1}+w_{2}\times {\text{FP}}_{2}\right)$$

where TP_1_, FN_1_, FP_1_ are the true positive, false negative, and false positive for the background category, while TP_2_, FN_2_, FP_2_ are the corresponding quantities for the neuropil category. Through this weighting approach, we increased the relative importance of the neuropils (as opposed to the background) in the loss function, thereby making the model more focused on accurately segmenting neuropils.

Categorical Focal Loss (CategoricalFocalLoss) is primarily suitable for addressing class imbalance in multi-class classification problems. It’s defined by


$$L\,\left(g_{t},p_{t}\right)=-g_{t}\cdot \alpha_{t}\cdot {\left(1-p_{t}\right)}^{\gamma }\cdot \log\left(p_{t}\right)$$

where *t* presents the class (neuropil or background), *g*_*t*_ = 1 if *t* is the ground truth class, *g*_*t*_ = 0 otherwise, *p_t_* is the predicted probability for the class *t*, α_t_ is the balancing factor for *t*, and γ is a parameter that adjusts the predicted probability.

For the background class, which accounts for the majority of the voxels, the model tends to predict a high probability *p_t_*. In this case, by increasing the γ parameter, we could reduce the loss contribution of these easily classified voxels. Conversely, for minority classes (neuropils), since *p_t_* was generally lower, CategoricalFocalLoss did not excessively reduce the loss for these samples. This operation balanced the loss between the background class and neuropils, reducing the impact of a higher number of predicted background class instances on the neuropil category, thereby enhancing the model’s ability to recognize imbalanced data.

In summary, the total loss function (Total Loss) is given by


Total⁢Loss=Weighted⁢Dice⁢Loss+(λ×CategoricalFocalLoss)

where λ is a hyperparameter used to balance the impact of the two loss functions, with a default value of 1, indicating equal consideration of both loss functions.

During the model training process, we set the batch size to 2 and the number of training epochs to 10. This ensured the model had sufficient data to learn from while avoiding overfitting. Additionally, to capture the model’s performance more accurately at different stages of training, whenever a lower loss value was observed on the validation set, we saved the model’s current state. Such a strategy allowed us to retain the best model during training and reduced the risk of model overfitting. By combining our chosen loss function, batch size, training epochs, and checkpoint monitoring strategy, we successfully designed a model training framework that converges quickly and has high generalization capabilities.

### 2.4 Comparative evaluation of segmentation algorithms

In our comparative evaluation, LYNSU was benchmarked against three other mainstream segmentation algorithms: Fully Convolutional Networks (FCN) (Long et al., 2014), 2D U-Net, and 3D U-Net. To ensure a fair comparison, all algorithms were trained and tested using the same dataset, implemented with identical training setups including the Adam optimization algorithm and the ReLU activation function for convolutional layers. The output layers across all models utilized a Softmax activation function to classify each pixel. For the architecture specifications, the FCN model was configured as FCN8s, which included five MaxPooling2D operations and three Conv2DTranspose layers for upsampling. The 2D U-Net architecture comprised four MaxPooling2D steps and UpSampling2D operations. Both 2D segmentation algorithms were trained using the Dice loss function. The training protocol was standardized across the FCN and 2D U-Net, with a batch size of 16 and a total of 5 epochs, balancing the need for sufficient training to capture complex features without overfitting given the limited epoch count.

For 3D U-Net, the training strategy mirrored the second stage of LYNSU, maintaining the same parameters and methods to ensure comparability. The primary difference lies in the initial approach: unlike LYNSU, 3D U-Net does not utilize the neuropil detection and localization stage. Instead, it directly processes the 3D images for segmentation. We applied the same Adam optimizer, ReLU activation in convolutional layers, and softmax output function as used in LYNSU’s second stage. This direct approach allows for a straightforward comparison of LYNSU and 3D U-Net segmentation efficacy, specifically focusing on their ability to delineate neuropil boundaries with or without the detection and localization stage.

### 2.5 Volume analysis and consistency assessment

To demonstrate a use case of the proposed LYNSU method, we analyzed the volume of left and right MB for 22,835 female *Drosophila* brains in the *FlyCircuit* database. We noted that many brain images in the database only cover partial brains. Therefore, for the purpose of the analysis, we implemented an automated filtering algorithm to select the images that had left and right MB being successfully segmented. Specifically, the algorithm used the measure module from the skimage library and scanned the segmented images to select those with exactly two closed 3D objects where the largest did not exceed twice the volume of the second. This condition effectively filtered our dataset to 10,337 brains suitable for manual verification. Note that this algorithm did not guarantee the selection of images with complete left and right MB. Images with partial left and/or right MB might be selected as well at this stage. In a later stage of the analysis, we visually inspected subsets of the images to ensure the completeness of the segmentation (see Results). For each selected brain, the volumes of the left and right MBs were calculated by counting the total number of voxels in the segmented volumes. The relative volume difference between the left and right MBs was computed using the formula:


Percent⁢volume⁢difference

$$=\left(\frac{\mathrm{Volume}\,\mathrm{of}\,\mathrm{Right}\,\mathrm{MB}-\mathrm{Volume}\,\mathrm{of}\,\mathrm{Left}\,\mathrm{MB}}{\mathrm{Average}\,\mathrm{Volume}\,\mathrm{of}\,\mathrm{Left}\,\mathrm{and}\,\mathrm{Right}\,\mathrm{MB}}\right)$$

A negative value indicates a larger left MB, whereas a positive value indicates a larger right MB. In the present study, we also analyzed the volume difference between the left and right hemispheres. We used a similar formula but replaced “MB” with “hemisphere.” The boundary between the left and right hemispheres was defined by a manually annotated midline in each brain image.

## 3 Results

**FIGURE 1:**
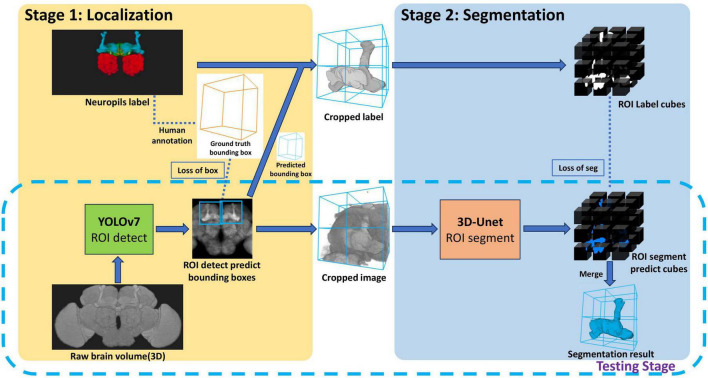
The LYNSU workflow. In Stage 1, rapid localization of neuropils is achieved using 2D brain projections and YOLOv7, resulting in the generation of ROIs encompassing complete neuropils. In Stage 2, 3D images are extracted from these ROIs and precisely segmented using the 3D U-Net model. The testing phase only required the workflow in the dashed box.

The proposed method, LYNSU, is designed based on a two-stage approach with the aim of achieving high-precision and high-speed brain segmentation (Figure 1). The goal of the first stage is to locate the target neuropils quickly and accurately. We first convert a 3D brain image stack into a 2D image by calculating the maximum and average brightness along the Z-axis direction, resulting in two 2D images. This ensures that the YOLOv7 model is exposed to a variety of brightness information in the images, enhancing its ability to identify the target neuropil. In the training phase, the YOLOv7 model is trained on human-labeled bounding boxes encompassing the target neuropil. In the test phase, the YOLOv7 model can rapidly generate a ROI containing the target brain neuropil for each 2D image. The goal of the second stage is to segment the target neuropil from the 3D image stack within the ROI generated from the first stage. In the graining phase, the 3D U-Net model is trained on human-segmented 3D image stacks. These 3D stacks are sliced into multiple cubes through an overlapping sliding method for data augmentation. We chose the 3D U-Net model for this delicate segmentation task, as this model can accurately capture the complex three-dimensional structure of neuropils and further enhance segmentation precision. Finally, the workflow achieves extremely high operational efficiency and accuracy in the testing phase.

**FIGURE 2:**
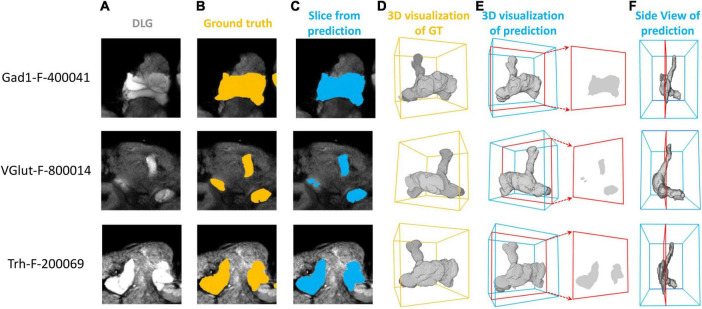
Segmentation of the MB (Mushroom Body) in three brain samples: Gad1-F-400041, VGlut-F-800014, and Trh-F-200069 (from top to down). **(A)** a slice of the anti-DLG image from the sample brain, **(B)** human segmentation as the ground truth, **(C)** segmentation by the proposed method (LYNSU), **(D)** 3D reconstruction of the ground truth segmentation, **(E)** 3D reconstruction of the LYNSU segmentation with the slice as shown in panel **(C)** and **(F)** the side view of the LYNSU segmentation. The red frames indicate the location of the slice. The display of different layer slices from these three MB samples showcases the precision of our segmentation method.

**FIGURE 3:**
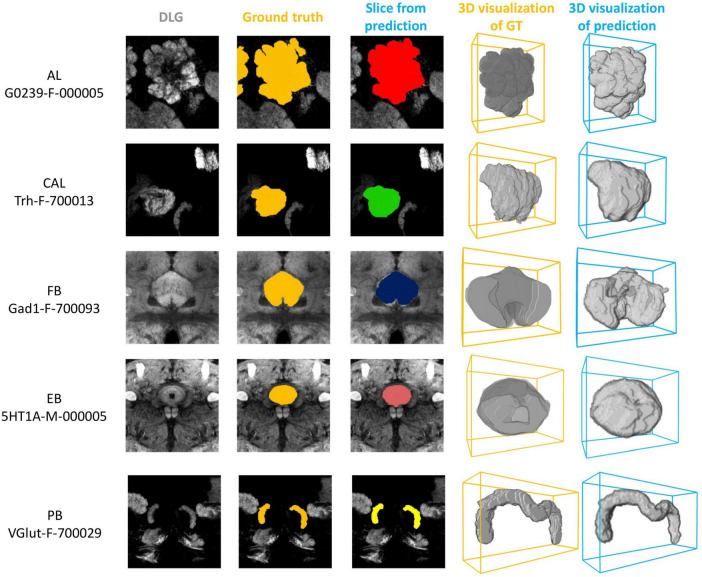
The segmentation results for five neuropils or brain structures: AL, CAL, FB, EB, and PB. From left to right: a slice of the anti-DLG image from the sample brain, the ground truth (GT), LNYSU segmentation, 3D reconstruction of GT and 3D reconstruction of LNYSU segmentation.

We first demonstrate the result of mushroom body (MB) segmentation with brain samples from three test sets: Gad1-F-400041, VGlut-F-800014, and Trh-F-200069. It is important to note that these three samples are 3D images not previously used by the model and come from different random splits of the dataset. We successfully achieved high-precision segmentation through the model’s inference process (Figure 2). These two-dimensional sections, compared to the corresponding ground truth, clearly demonstrate that the MB in three different brain samples were precisely segmented by LYNSU. We further demonstrate the segmentation results of five other neuropils or brain structures, AL (Antennal Lobe), CAL (Calyx), FB (Fan-shaped Body), EB (Ellipsoid Body), and PB (Protocerebral Bridge), from different sample brains in the test set (Figure 3).

**FIGURE 4:**
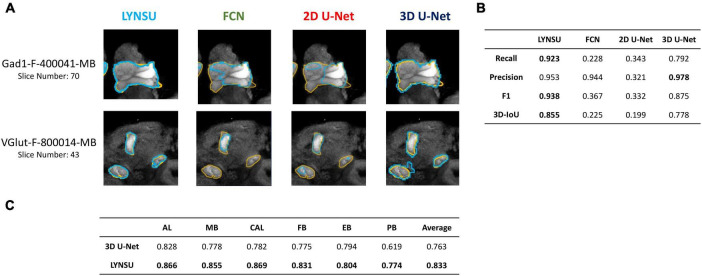
Performance comparison between LYNSU, FCN, 2D U-Net, and 3D U-Net based on the images from the *FlyCircuit* dataset. **(A)** Selected 2D slices from two sample brains (top and down) demonstrate superior boundary delineation by LYNSU. Orange: ground truth. Blue: predicted boundaries by each algorithm. **(B)** Quantitative metrics, including Recall, Precision, F1, and 3D-IoU, indicate that LYNSU’s performance is much better than that of other algorithms. **(C)** 3D IOU scores for the segmentation of six neuropils or brain structures, compared between LYNSU and 3D U-net (without YOLO) on the test set. The former outperform the latter in all tests.

We systematically compare LYNSU to three other mainstream segmentation algorithms without using YOLO: FCN, 2D U-Net, and 3D U-Net. First, visual inspection of the 2D slices indicates that LYNSU produces more precise neuropil boundaries than other algorithms (Figure 4A). Next, we conducted a quantitative evaluation and using four commonly used metrics: Recall, Precision, F1 score, and 3D-IoU. The LYNSU model outperformed the other three algorithms (Figure 4B). Specifically, the LYNSU model performs much better than FCN and 2D U-Net by a large margin in all metrics and is also much better than 3D U-Net in three out of four metrics. We conducted ten random splits of the dataset, with 16 brains assigned as the training set and two brains as the test set. We test the performance of LYNSU against 3D U-net (without YOLO) for all six neuropils or brain structures (AL, MB, CAL, FB, EB, PB), and LYNSU achieve a higher 3D IOU score than 3D U-net for all neuropils or brain structures (Figure 4C) with an average 3D IOU exceeding 0.833 compared to 0.763 for 3D U-net. These results strongly validate the capability of LYNSU to segment various brain regions of the *Drosophila* brain.

**FIGURE 5:**
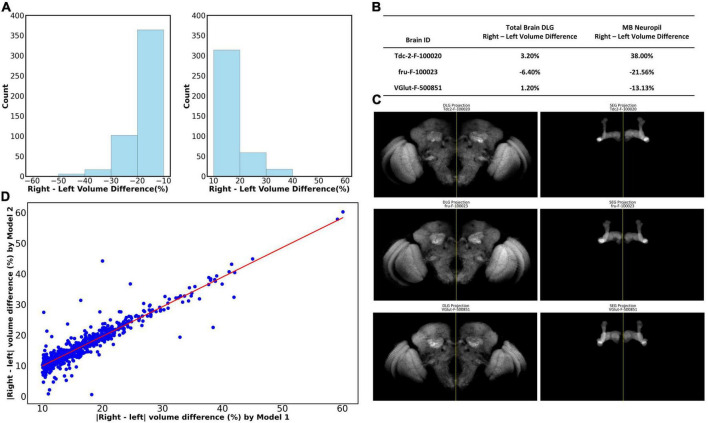
The volumetric asymmetry of the MB was revealed by LYNSU. **(A)** The distribution of volume differences between the right and left MB for which the differences are larger than 10%. These brains account for 10.14% of the parent group under analysis. (Left) Left volume > right volume. (Right) Right volume > left. **(B)** Analysis showing that the large left and right MB volume differences cannot be attributed to the bilateral brain volume difference. We analyzed three sample images, Tdc2-F-100020, fru-F-100023, and VGlut-F-500851, which cover the complete brain volume. The left and right hemisphere volumes of the sample brains do not exceed 6.4%, while the MB volume differences are all larger than 13%. **(C)** (Left) The whole brain images for the three brains listed in **(B)**. Note the symmetry between the left and right hemispheres. The yellow vertical lines are the midlines. (Right) The MBs segmented by LYNSU from the brain shown in the left. Note the apparent asymmetry between the left and right MBs. **(D)** The highly consistent inference of the volume by LYNSU. We compare the left-right MB volume difference inferred by Model 1 (*x*-axis) and Model 2 (*y*-axis). Both are LYNSU but are only trained with different training sets. Each data point (blue dot) on the plot corresponds to a brain image. The red line (slope = 0.97) depicts the linear regression of the data.

We further demonstrate one potential application of LYNSU by studying the asymmetry of Drosophila brains. We ask whether the left and right mushroom bodies are of the same size at the population level. Without the automated segmentation algorithm, answering this seemingly simple question requires an unrealistic amount of human labor for manual segmentation. Here, we applied LYNSU to the entire dataset of female *Drosophila* brains in the *Flycircuit* database, which hosts 22,835 female brains. To evaluate the segmentation accuracy, we employed an automated filtering algorithm to quickly select 10,337 brain images that contain both left and right MBs. From this subset, we randomly sampled 1,000 brains for manual verification and revealed an accuracy of 98.8% in segmenting the MB neuropil; only 12 brains were identified as segmentation failures. We further calculated the volumes (number of voxels of the segmented region) of the left and right MBs in each brain and identified 2,404 brains that possess large bilateral differences (> 10% difference between left and right). We noted that some of these large bilateral differences are caused by incomplete left and right MB coverages in the images. These images are considered invalid samples and have to be excluded from the analysis. Manual verification identified 883 out of the 2,404 brains as valid (Figure 5A). Note that the original 10,337 brains also contain invalid samples, but the number is too large for manual inspection. Instead, we manually inspected the aforementioned 1000 random samples and identified that 84.2% (842 of 1,000) of them are valid. By assuming that 84.2%, or 8,703, of the 10,337 brains are valid, we can conclude that 10.14% (883/8,703) of the fly brains have MB volumetric asymmetry exceeding 10%.

We ask whether the large volume differences between the left and right MBs are due to unexpected unilateral deformation of the brain during the sample preparation and imaging processes. Although most of the brain images in the *FlyCircuit* database do not cover the whole brain, we still identified several images that contain the complete brain volume. We visualize three of these sample brains that have large MB volume asymmetry (> 10%) (Figures 5B, C). By analyzing the volume of the left and right hemispheres of these brains, we found that they are highly symmetric, and the volume differences between the two hemispheres are merely 1.2% to 6.4%. The result showed that the MB volume asymmetry is not simply the reflection of the asymmetry of the entire brain.

Finally, we would like to make sure that the MB asymmetry is not a model-specific artifact. To this end, we trained another LYNSU model (Model 2) with a different training set and analyzed the 883 brain images with large MB asymmetry selected by the original model (Model 1) described above. The volumetric discrepancies between the segmented results from the two models and the Ground Truth were minimal, at 1.19% and 2.16%, respectively. This finding substantiates that differences in the volume of left and right MB greater than 3% are not likely attributable to modeling errors. To further validate the stability and consistency of our models, we compare the inferred left-right MB volume differences of the 883 brains between the two models. We found that the volume differences inferred by the two models are highly consistent, and a linear regression of the data yields a slope of 0.97, suggesting strong correlation and model reliability (Figure 5D). This evidence underscores our models’ capability to reliably segment complex neural structures with high precision.

## 4 Discussion

This study successfully developed a novel, efficient, and accurate LYNSU segmentation workflow, specifically for neuropil segmentation in fluorescence images of *Drosophila* brains. By combining YOLOv7 and 3D U-Net, LYNSU substantially outperformed FCN and 2D U-Net, which were inadequate for the task. LYNSU also exhibited marked improvement over 3D U-Net by up to 15.5% in 3D IoU. It efficiently segmented a neuropil in just 7 s, demonstrating its suitability for large-scale databases.

In the present study, we trained and validated individual YOLO models for each type of neuropil. However, to improve the efficiency, it is possible to integrate the localization labels of all neuropils to train a single YOLO model. We have performed a preliminary test on this idea. We first balanced the training set to ensure the model did not favor specific object labels. We integrated the localization labels of all neuropils to train a single model. The training set was balanced to ensure the model did not favor any specific object labels, allowing it to simultaneously detect AL, MB, and the Central Complex regions (including FB, EB, and PB). Our preliminary test achieved 100% accuracy on the test set for these regions. This approach not only maintained a high level of accuracy but also significantly reduced the computational time required for inference. We may implement the YOLO model integration in the next version of LYNSU.

A recent study (Iriawan et al., 2024), demonstrated a similar concept by employing YOLOv3-v4 and 2D U-Net to segment the MRI images of human brains. It was a relatively small-scale study with a total of 346 2D MRI images for training/validation and merely 14 images for testing. We tested YOLOv4 and 2D U-Net and FCN in the early stage of the study [presented on September 3^rd^, 2022 at the Taiwan Society for Neuroscience International Conference: (Poster Number: P2-41)]^2^ and were not satisfied with the performance. Therefore, we changed to YOLOv7 and 3D U-Net as described in the present study for better performance. We also performed a larger scale training/validation with 2,060 image slices (from 18 brains) and a much more thorough and rigorous test with ∼100,000 slices from 1,000 brains.

This new method can potentially solve pressing issues in current connectomics, especially regarding spatial errors and computational efficiency in image alignment and registration. Previous methods often required aligning images using a standard brain template. Although convenient, these methods unavoidably introduce spatial errors. Alternatively, one can segment neuropils or brain structures manually for individual images without using the standard brain template to achieve higher spatial accuracy. However, manual segmentation is extremely time-consuming and is not suitable for large-scale studies. In contrast, our algorithm combines the advantages of both approaches and achieves high spatial accuracy and temporal efficiency. Specifically, our algorithm can complete a neuropil segmentation task in just 7 s, which would take a human expert 4 h. This breakthrough is significant considering the need for high-throughput connectomics research and will significantly accelerate the entire research process.

LYNSU has the potential to be generalized to brain images of other insect species captured using confocal microscopy with fluorescent anti-DLG staining. However, new species and neuropils would require the preparation of corresponding labeled data, which can be time-consuming and resource-intensive. Applying LYNSU on mammalian brains with different staining technologies is possible, but it may require extensive testing and tweaking due to the distinct anatomical features between mammals and insects.

LYNSU offers several important applications, enhancing our understanding of neuroanatomy at an individual level. Using LYNSU, we have identified that over 10% of female Drosophila exhibit significant volumetric differences between the left and right MB neuropils—a phenomenon that was previously difficult to quantify accurately in large-scale studies due to limitations associated with image warping and registration. Our findings pave the way for future studies to explore the variability of neuropil morphology among individuals more accurately. Furthermore, LYNSU enables more precise quantification of gene distribution across different neuropils, which is not feasible with traditional methods that rely on warping and registering images to a standard brain template. This capability is crucial for advancing our understanding of functional neuroanatomy and could lead to significant breakthroughs in connectomics research, facilitating a deeper exploration of individual differences and their genetic underpinnings in neuropil structure and function.

## Data availability statement

The original contributions presented in this study are included in this article/supplementary material, further inquiries can be directed to the corresponding authors.

## Ethics statement

The manuscript presents research on animals that do not require ethical approval for their study.

## Author contributions

K-YH: Investigation, Methodology, Software, Validation, Visualization, Writing−original draft. C-TS: Conceptualization, Funding acquisition, Supervision, Writing−review and editing. N-YC: Conceptualization, Funding acquisition, Supervision, Writing−review and editing. C-CL: Conceptualization, Funding acquisition, Supervision, Writing−review and editing, Writing−original draft.

## Footnotes

1
http://www.flycircuit.tw

2
https://tsfn.neuroscience.org.tw/pastMeetingIn/2022
